# Using dried blood spots collected under field condition to determine HIV-1 diversity and drug resistance mutations in resource limited Tanzania

**DOI:** 10.7448/IAS.17.4.19686

**Published:** 2014-11-02

**Authors:** James Kimaro, Elichilia Shao, Balthazar Nyombi, Emanuel Kifaro, Dorcas Maruapula, Simani Gaseitsiwe, Rosemary Musonda

**Affiliations:** 1 Clinical Laboratory, Kilimanjaro Christian Medical Centre, Moshi, Tanzania; 2 HIV Research Lab, Botswana Harvard HIV Research Lab, Gaborone, Botswana

## Abstract

**Introduction:**

A dried blood spot (DBS) on filter paper has been used for different tests globally and has gained popularities in resource limited settings especially during HIV/AIDS epidemic. We assessed the efficiency of molecular characterization of HIV-1 subtypes using DBS collected under field conditions in northern Tanzania.

**Materials and Methods:**

In 2011 and 2012, 60 DBS samples were collected under field conditions from exposed and newly diagnosed HIV-1 infected children from Kilimanjaro (n=20), Arusha (n=20), Tanga (n=10) and Manyara (n=10).

**Results and discussion:**

Of 60 DBS analyzed at both Protease (PR) and Reverse Transcriptase (RT) regions, 45 (75%) were analyzed, including 17 (85%) from Kilimanjaro, 15 (75%) from Arusha, 8 (80%) from Tanga, and 5 (50%) from Manyara region. All 45 DBS characterized had viral load above 1000 copies/mL with mean log10 viral loads of 3.87 copies/mL (SD 0.995). The phylogenetic results indicated presence of subtype and circulating recombinant form (CRF). In which, 24 were subtype A1 (53.33%), 16 were subtype C (35.55%), 3 were subtype D (6.67%) and 2 were CRF10_CD (4.35%). All major mutations were detected in the RT region, none from protease (PR) region. The mutations detected were Y181C (n=8), K103 (n=4) and G190A (n=1), conferring resistance to non-nucleoside reverse transcriptase inhibitors (NNRTIs), and M184V (n=1), conferring resistance to lamivudine and emtricitabine.

**Conclusions:**

Our results indicate that DBS collected from field conditions in resource scarcity areas can be used to determine the phylogeny of the virus and drug resistance mutations in areas with diverse HIV-1 group M subtypes.

